# Supplementary material for: High-Risk Clone of Klebsiella pneumoniae Co-Harbouring Class A and D Carbapenemases in Italy
Source: Int J Environ Res Public Health. 2022 Feb 24;19(5):2623. doi: 10.3390/ijerph19052623 (PMC8909938; doi:10.3390/ijerph19052623)
Supplement: Supplementary file 1 [file ijerph-19-02623-s001.zip › Figure S1.pdf]

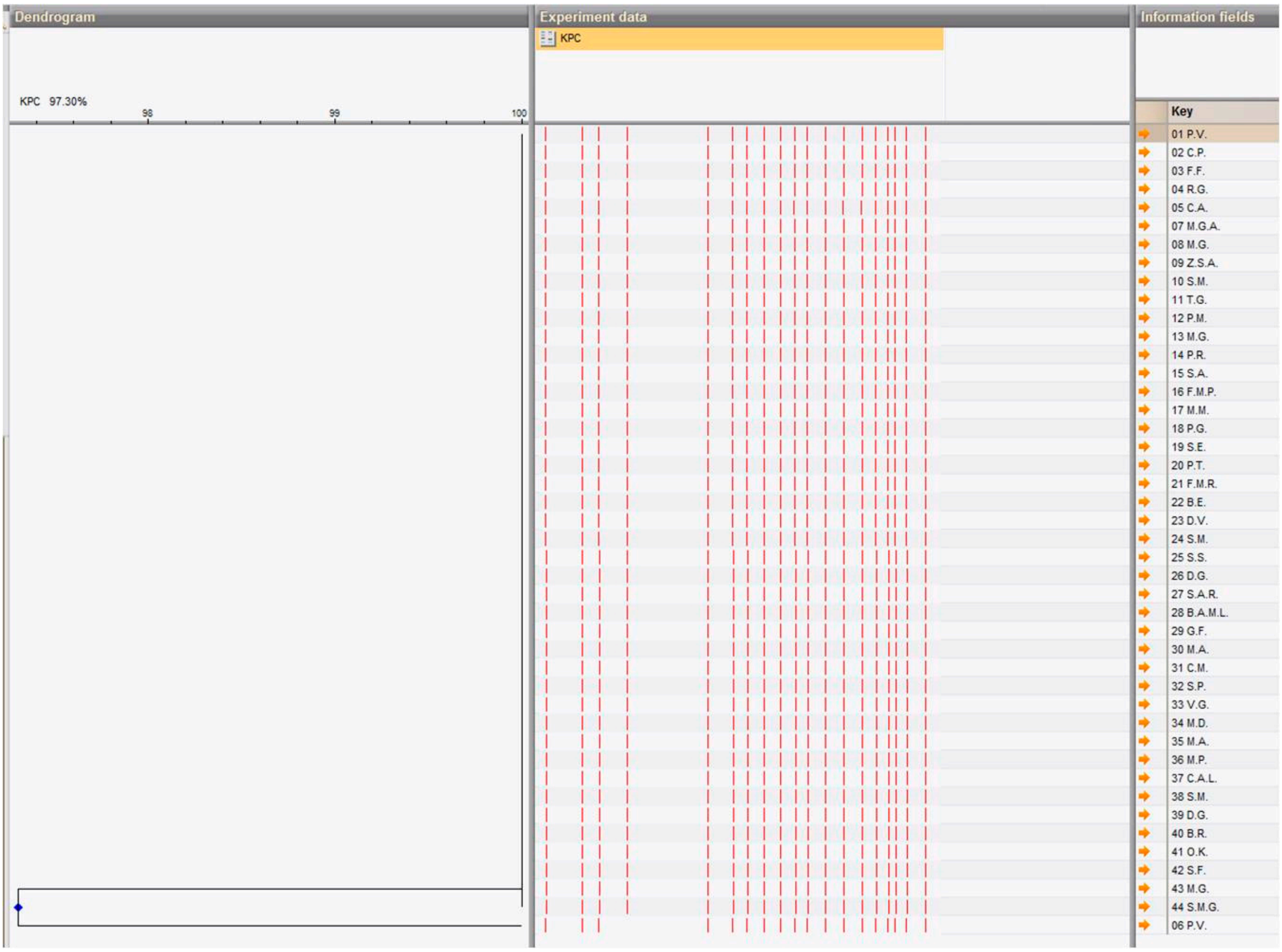

Figure S1: Dendrogram and electrophoretic bands of *Klebsiella pneumonia* isolates, co-producing KPC and OXA-48 carbapenemases.
