# Supplementary material for: High-Risk Clone of Klebsiella pneumoniae Co-Harbouring Class A and D Carbapenemases in Italy
Source: Int J Environ Res Public Health. 2022 Feb 24;19(5):2623. doi: 10.3390/ijerph19052623 (PMC8909938; doi:10.3390/ijerph19052623)
Supplement: Supplementary file 1 [file ijerph-19-02623-s001.zip › S2.pdf]

S2: MLST analyses: Gene included in the *Klebsiella pneumoniae* MLST analyses, PCR and sequencing primers and amplification condition:

Housekeeping genes:

rpoB (beta-subunit of RNA polymerase)

gapA (glyceraldehyde 3-phosphate dehydrogenase)

mdh (malate dehydrogenase)

pgi (phosphoglucose isomerase)

phoE (phosphorine E)

infB (translation initiation factor 2)

tonB (periplasmic energy transducer):

Primers:.

Gene	**Primers (F+R)**
rpoB	F: Vic3: GGCGAAATGGCWGAGAACCA<br>R: Vic2: GAGTCTTCGAAGTTGTAACC
gapA	F:gapA173: TGAAATATGACTCCACTCACGG<br>R:gapA181: CTTCAGAAGCGGCTTTGATGGCTT
mdh	F:mdh130: CCCAACTCGCTTCAGGTTCAG<br>R:mdh867: CCGTTTTTCCCCAGCAGCAG
pgi	F:pgi1F: GAGAAAAACCTGCCTGTACTGCTGGC<br>R:pgi1R: CGCGCCACGCTTTATAGCGGTTAAT
phoE	F:phoE604.1: ACCTACCGCAACACCGACTTCTTCGG<br>R:phoE604.2: TGATCAGAACTGGTAGGTGAT
infB	F:infB1F: CTCGCTGCTGGACTATATTCG<br>R:infB1R: CGCTTTCAGCTCAAGAACTTC
tonB	F:tonB1F: CTTTATACCTCGGTACATCAGGTT<br>R:tonB2R: ATTCGCCGGCTGRGCRGAGAG

Mix PCR:.

**Reagents**	**Quantity (µl)**
Mastermix	9.3
H_2O	10
$MgCl_2$	1.2
Primers F+R	1.5
Samples	3

Amplification:.

**94 °C**	2 mins	
**94 °C**	30 sec	
**Temperature gradient ***	30 sec	x30 cycles
**72 °C**	1 min	
**72 °C**	5 mins	

* *tonB*: 47.5 °C; *mdh*:53 °C; *pgi, phoE, infB*: 50 °C; *gapA*: 60.5 °C; *rpoB*: 55 °C.
